# Liposomal anesthetic gel for pain control during periodontal therapy in adults: a placebo-controlled RCT

**DOI:** 10.1590/1678-7757-2019-0025

**Published:** 2019-11-11

**Authors:** Gustavo Simao Moraes, Isadora Benato dos Santos, Shelon Cristina Souza Pinto, Marcia Thais Pochapski, Paulo Vitor Farago, Gibson Luiz Pilatti, Fabio Andre Santos

**Affiliations:** 1 Universidade Estadual de Ponta Grossa, Departamento de Odontologia, Ponta Grossa, Paraná, Brasil.; 2 Universidade Estadual de Ponta Grossa, Departamento de Ciências Farmacêuticas, Ponta Grossa, Paraná, Brasil.

**Keywords:** Anesthetics, Periodontal debridement, Pain management, Dental scaling, Clinical trial

## Abstract

Periodontal therapy usually requires local anesthesia. If effective, a non-invasive, liposomal anesthetic gel could increase the levels of acceptance of patients in relation to periodontal therapy.

**Objective:**

This study investigated the efficacy of liposomal anesthetic gel for pain control during periodontal therapy.

**Methodology:**

Forty volunteers with moderate to severe chronic periodontitis were recruited, of which at least three sextants required periodontal therapy. At least one of the selected teeth had one site with a probing depth of ≥4 mm. The volunteers received the following three gels: a placebo, lidocaine/prilocaine (Oraqix^®^), or a liposomal lidocaine/prilocaine, which were applied to different sextants. Pain frequency was registered during treatment and the volunteers received a digital counter to register any painful or uncomfortable experiences. At the end of each session, the volunteers indicated their pain intensity using rating scales (NRS-101 and VRS-4). The volunteers had their hemodynamic parameters measured by a non-invasive digital monitor.

**Results:**

Pain frequency/intensity did not show statistical difference between intervention groups. The tested gels did not interfere with the hemodynamic indices. Dental anxiety, suppuration and probing depth could influence pain during periodontal therapy.

**Conclusion:**

Our results suggest limited indications for the use of non-invasive anesthesia when used for scaling and root planing. Intra-pocket anesthetic gel could be a good option for anxious patients, or those who have a fear of needles.

## Introduction

Severe periodontitis is the sixth most prevalent condition worldwide, affecting 11% of the global population (743 million people aged 15-99 worldwide). It has remained static on a global scale during the last two decades. The age-standardized incidence of the disease did not change from 1990-2010 (696 and 701 cases per 100,000 person-years, respectively). These prevalence and incidence rates were similar for males and females, and increased with age, with a steep increase among individuals aged from 30 to 40 years old, remaining stable thereafter.^1^

Periodontal therapy usually involves supra and/or subgingival scaling and root planing (SRP),^2^ which can be performed using periodontal curettes or sonic and ultrasonic instruments. The prevalence of pain and discomfort during SRP is variable, but studies report around 15% to 33% of patients describing it as a significantly painful experience.^3^^,^^4^ Reasons for this include tissue trauma caused by instrumentation, dentin hypersensitivity,^2^ and the unpleasant noise and sensation produced by periodontal curettes and sonic or ultrasonic instruments when they come into contact with the tooth structure.^5^^,^^6^

The usual technique to control pain and discomfort during SRP is applying local anesthetic injections (nerve block or infiltration anesthesia).^7^^–^^9^ However, patients often report a fear of needles, and complain about pain and discomfort caused by their insertion and prolonged numbness in the surrounding soft tissues.^7^^,^^8^^,^^10^^,^^11^ These factors can lead patients to delay, or even avoid, periodontal therapy.^7^^,^^12^

New anesthetic formulations have been developed to improve treatment conditions and ameliorate the patients' level of acceptance of dental procedures.^11^^,^^13^ Oraqix^®^ (25 mg/g lidocaine and 25 mg/g prilocaine) was developed with the addition of a thermosetting agent.^9^^,^^14^^,^^15^ The onset of anesthesia has been shown to range from 30 seconds to 2 minutes after its application, and some studies have demonstrated that it is the best non-invasive anesthesia option for SRP thus far.^8^^,^^11^^,^^14^^,^^16^ Another alternative is the combination of local anesthetics with liposomal formulations, which can increase the duration of anesthesia, decrease central nervous and cardiac toxicity, and decrease circulating plasma levels.^17^^–^^19^ Studies have shown significant skin and oral mucosa anesthesia using liposome-encapsulated anesthetics;^20^^–^^22^ but until now, there has been no evaluation of their efficacy in the periodontal pocket, especially during SRP.

The aim of this study was to compare the effects of a liposomal, lidocaine/prilocaine, thermosetting anesthetic gel for pain control during scaling and root planing (anti-infective periodontal therapy) compared to Oraqix^®^ (positive control) gel, and a placebo gel (negative control). Our primary outcome was the frequency/intensity of pain, obtained by using a digital counter to register any painful experience; a numerical rating scale (NRS-101) and a verbal rating scale (VRS-4) were used. The secondary outcomes were hemodynamic parameters (systolic blood pressure, diastolic blood pressure, heart rate and oxygen saturation). The null hypothesis was that there would be no difference between intervention groups (placebo and anesthetic gels) in relation to pain control during scaling and root planing.

## Methodology

### Study population

**Figure 1:**
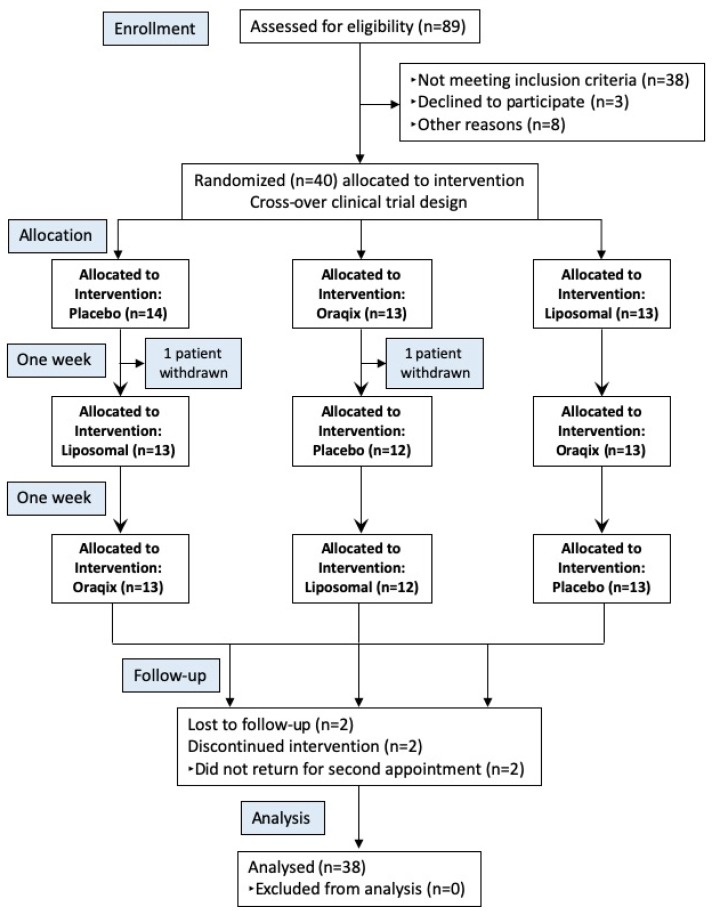
Flowchart of subjects throughout the stages of the study. At the end of the clinical trial, all untreated sextants received periodontal treatment

A flowchart of the overall study design is shown in Figure 1. After approval by the Joint Research and Ethics Committee (CEP – 78.2009.15036.09; Clinical Trials Registry: Primary Id Number: RBR-934sys), we selected forty volunteers for this randomized, double-blind, cross-over, placebo-controlled clinical trial. The volunteers were individually made aware of the study protocol and the aims of the study prior to enrollment. The study was performed according to the guidelines of the Helsinki Declaration.

### Sample size

Sample size calculation was based on pain intensity (primary outcome) using visual analogue scale (VAS) data that was previously published in a study which reported the effects of intra-pocket lidocaine and prilocaine gel (2.5% each) in SRP.^13^ If the sample size in each intervention group was 38 (1:1:1 allocation ratio), a two-sided test would have 80% power at an effect size of 0.65, and 0.05 significance level in order to detect a minimum, clinically important difference of 15 units in the VAS. The sample size was increased to 40 volunteers per group to account for the potential loss of volunteers during the study. Sample size was calculated using a specific software (G*Power Version 3.1.9.2; http://www.gpower.hhu.de).

### Randomization and allocation concealment

Block randomization was used to allocate three sextants of each volunteer in relation to the interventions (placebo, Oraqix^®^ and Liposomal), using a computer program (Microsoft® Excel for Mac version 10.4, 2011). The random allocation sequence was printed and sealed in envelopes with the volunteer's number on the outside. This procedure was performed and monitored by one researcher (FAS).

### Inclusion and exclusion criteria

The volunteers had to be aged 18 or older; they needed anti-infective periodontal therapy and had to have at least three sextants with a minimum of two vital teeth requiring scaling and root planing. They also needed to have sought dental care at the University Dental Clinic. At least two sites per sextant with a probing depth of 4 mm or more and clinical signs of periodontal disease (presence of supra/subgingival calculus and/or dental biofilm, as well as bleeding/suppuration on probing) were required for them to be included in this study.

The exclusion criteria were: individuals undergoing periodontal maintenance; any allergic reaction to amide anesthetic; consumption of any analgesic or anti-inflammatory drugs in the 12 hours prior to treatment; individuals with a history of alcoholism; smokers; pregnant or lactating women; individuals with any non-controlled systemic disorder such as cardiac, neurological, kidney, liver, hematological or psychological alterations; individuals with presence of ulcerative lesions, abscesses or acute infections; individuals with the need for dental extraction in the selected sextants; individuals with dentin hypersensitivity; and individuals with endodontic treatment or any other alteration that could compromise the measurement of data.

### Evaluation of clinical parameters

The data regarding probing depth, width of keratinized mucosa, bleeding on probing (presence or absence), dental plaque (presence or absence) and suppuration (presence or absence) were obtained at baseline. Measurements were performed using a standardized UNC periodontal probe (Hu-Friedy^®^, Rio de Janeiro, RJ, Brazil). The volunteers were also asked to complete Corah's Dental Anxiety Scale (DAS) before the first treatment session.

### Anesthetic procedures and treatment

Three different gels were used in this study: a lidocaine/prilocaine thermosetting gel (Oraqix^®^, Dentsply, York, PA, United States), which served as positive control, a liposomal lidocaine/prilocaine thermosetting gel (granulated lecithin (6.6 g); isopropyl palmitate (7.6 mL); sorbic acid or benzoic acid (0.12 g); poloxamer 407 (6.78 g); poloxamer 188 (2.40 g); potassium sorbate or sodium benzoate (0.1 g); purified water (30 mL on the first day/10 mL on the second day); ethanol (2.5 mL); hydrochloric acid (0.1 mL); lidocaine (25 mg/g) and prilocaine (25 mg/g)). The large multilamellar liposomes were obtained by the thin film hydration method. Subsequently, lecithin and isopropyl palmitate were dissolved in chloroform. The lipid mixture was then deposited as a thin film into a round-bottomed flask using a rotary evaporator after full removal of the chloroform under vacuuming at 40°C in a temperature-controlled water bath for a period of 2 hours to ensure the absence of solvent residue. The films were suspended in 20 mmol/L HEPES buffer (7.4 pH, containing 154 mmol/L NaCl), and multilamellar vesicles were obtained after vortexing at room temperature (5 min, 25°C). Large multilamellar vesicles were prepared by extruding (20 cycles) these vesicles within 400 nm-membrane filters at 25°C using an extruder device (Lipex Biomembranes Inc. Vancouver, BC, Canada). The loaded liposomes containing lidocaine base (2.5%) and prilocaine base (2.5%) were prepared by adding these anesthetics directly to the previously obtained liposomes after extrusion at the final concentration of 5%. The loaded liposomes formed appear as concentric and non-concentric multilamellar vesicles by TEM with 327.5±17.5 nm and PDI of 0.21±0.08 by dynamic light scattering. The mean encapsulation efficiency was 54.9±15.2% by HPLC/DAD. The unloaded and loaded formulations were stable at 4°C for 3 months. The thermosetting gel was prepared using the poloxamer solutions (407 and 188). These polymers were dispersed in purified water via manual stirring. The solutions were then stored in a refrigerator at 4°C for at least 12 h to ensure complete solvation. Liposomal anesthetics were then added to the gel with manual stirring until a homogeneous dispersion was obtained. The placebo gel had the same composition as the liposomal gel, but without the anesthetic bases, and served as negative control. All the selected volunteers received the gels on different teeth, from different sextants, using a cross-over design and with one-week intervals between each appointment to avoid cross-over contamination (spill-over effect).^23^ The anesthetic agents were stored in identical syringes and were identified only by letters. The anesthesia was administered by a blinded operator (SCSP) according to a randomization process, who also performed the periodontal therapy procedures. The sextants were isolated with cotton rolls and the anesthetic gel was placed inside the periodontal pocket for about one minute before SRP. A second examiner (GSM) was responsible for collecting the data of each volunteer. SRP was performed using Gracey and McCall curettes (Millennium^®^, Golgran, Rio de Janeiro, RJ, Brazil). The volunteers could ask for rescue anesthesia (3% prilocaine with 0.03 IU/mL felypressin injection, Citanest^®^, Dentsply, Catanduva, SP, Brazil) if they were still feeling pain after two applications of gel.

### Pain evaluation

Pain frequency was obtained during the SRP procedures; the volunteers received a digital counter to register any painful or uncomfortable experience. At the end of each session, the volunteers were asked to indicate pain intensity using the numerical rating scale (NRS-101), and discomfort using a four-point verbal rating scale (VRS-4) (1=none; 2=mild; 3=moderate; 4=severe). If rescue anesthesia was required, the pain and discomfort scores were obtained before it was administered. The volunteers were also asked to rate how unpleasant the taste of the anesthetic agent was (“acceptable”; “slightly unpleasant”; “very unpleasant” and “I would not like to receive it again”).

### Assessment of hemodynamic parameters

During the treatments, the volunteers had their hemodynamic parameters (systolic blood pressure, diastolic blood pressure, heart rate and oxygen saturation) measured by a non-invasive digital monitor (INMAX^®^ Monitor Multiparamétrico, Instramed Indústria Médico Hospitalar Ltda, Porto Alegre, RS, Brazil).

### Statistical analysis

The data regarding pain frequency and the NRS-101 and VRS-4 pain scales did not show normal distribution and homogeneity of variance. The volunteers had different sextants treated with different numbers of teeth in each one, so a paired test would not be suitable; the Kruskal-Wallis non-parametric test was used to determine the differences between intervention groups.

The clinical parameters, such as distribution of sextants, probing depth (≤3 mm, 4-5 mm or 6 mm) and keratinized mucosa (≤2 mm or >2 mm), were compared using the chi-square test (χ^2^). The mean number of teeth, dental plaque, bleeding on probing, suppuration, probing depth and width of keratinized mucosa were analyzed by the Kruskal-Wallis test (non-normal distribution).

A univariate regression analysis, with random intercept, was performed to explore the relationship between the primary outcome of interest (pain frequency/intensity) and the various risk factors^24^, comprising the following levels: site level (dental plaque, bleeding on probing, suppuration probing depth and width of keratinized mucosa), sextant level (position and number of teeth) and subject level (age, gender, periodontal diagnosis and dental anxiety).

The hemodynamic parameters (systolic and diastolic blood pressure, heart rate and oxygen saturation) were analyzed at baseline, during and after treatment by repeated measures ANOVA and the paired t-test.

The data obtained via trans-operative and post-operative scaling and root planing in the three intervention groups, such as volume of anesthetic gel applied, number of applications, need for additional (rescue) anesthesia, time required for periodontal therapy (per sextant), difficulties during treatment, post-operative discomfort due to treatment and the volunteers' perceptions of the gel's flavor were analyzed by the ANOVA, Kruskal-Wallis and χ^2^ tests, according to the type and distribution of the variables.

The tests were considered statistically significant when p<0.05 (IBM^®^ SPSS^®^ 21.0 Statistics, IBM Corp., Armonk, NY, USA).

## Results

Of the forty randomized volunteers, thirty-eight managed to conclude the study and two did not return for the second appointment. Figure 1 shows the study flowchart. The sample comprised 23 (60%) females and 15 (40%) males, aged from 26 to 73 years old (mean age: 43.6±11.2 years old) and diagnosed with localized periodontitis (45%) or generalized periodontitis (55%).

**Figure 2:**
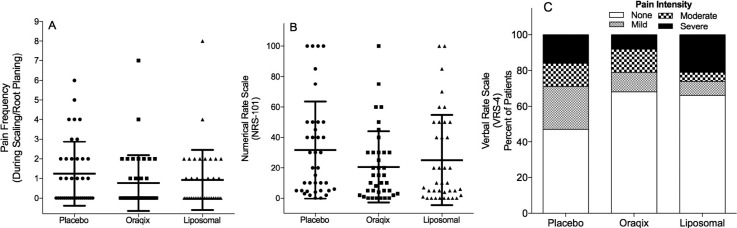
Pain during/after scaling and root planing in periodontal therapy. (A) Median with interquartile range (lines) of pain frequency (no significant findings, p=0.316). (B) Median with interquartile range (lines) of NRS-101 (no significant findings,p =0.250). The dots correspond to each volunteer. (C). VRS-4 scores: Percentage of subjects in the Placebo, Oraqix^®^; and Liposomal groups who reported no, mild, moderate, and severe pain (no significant findings, p=0.231). Kruskal-Wallis test

Figure 2 shows the pain felt during/after SRP by the different groups using pain frequency and the numerical rating scale (NRS-101) and verbal rating scale (VRS-4) values. The number of volunteers reporting no pain frequency during SRP was 19 (50%) for the placebo; 25 (66%) for Oraqix^®^, and 21 (55%) for liposomal gel. No statistically significant differences were found between intervention groups (p>0.05). The mean ±SD (95% CI) values for NRS-101 were 31.6±31.9 (21.1 – 42.1) for the placebo; 20.5±23.5 (12.8 – 28.2) for Oraqix^®^; and 25.0±29.6 (15.3 – 34.7) for liposomal gel. The number of volunteers reporting no pain (pain scale=0) using the NRS-101 scale was two (5%) for the placebo; six (16%) for Oraqix^®^; and eight (21%) for liposomal gel. Using the VRS-4 scale, no pain was observed in 18 (47%), 26 (68%) and 25 (66%) volunteers for those who underwent SRP with the placebo, Oraqix^®^, and liposomal gel, respectively. No statistical differences were found between intervention groups (p<0.05).

The sextants, number of teeth, and clinical parameters (dental plaque, bleeding on probing, suppuration, probing depth, and width of keratinized mucosa) are shown in Table 1. The intervention groups showed statistically significant differences (p<0.0001) for suppuration. Sextant position, number of teeth, dental plaque, bleeding on probing, probing depth, and width of the keratinized mucosa had a similar distribution, with no statistically significant differences (p>0.05).

**Table 1:** Distribution of sextants, number of teeth and periodontal clinical parameters: Dental plaque (DP); Bleeding on Probing (BoP); Suppuration; Probing depth (PD) and width of keratinized mucosa (KM)

Parameters	Intervention Groups									p
	Placebo			Oraqix^®^			Liposomal			value
Sextants (%)*										0.834ns
Upper Right	6 (16)			5 (13)			9 (24)			
Upper Anterior	5 (13)			8 (21)			4 (11)			
Upper Left	10 (26)			6 (16)			6 (16)			
Lower Left	5 (13)			4 (11)			3 (8)			
Lower Anterior	5 (13)			6 (16)			8 (21)			
Lower Right	7 (18)			9 (24)			8 (21)			

Number of teeth†	121			121			114			0.580ns
Mean±SD	3.2±0.9			3.2±1.0			3.0±1.0			
Median (IqR)	3.0 (2.0 – 4.0)			3.0 (2.8 – 4.0)			3.0 (2.0 – 4.0)			

DP (%)†										0.777ns
Mean ±SD	65.7±32.1			72.4±25.3			69.3±31.0			
Median (IqR)	75.0 (37.5 – 100)			75.0 (50.0 – 93.8)			79.2 (50.0–100)			

BoP (%)†										0.235ns
Mean ±SD	89.8±18.2			82.0±26.0			89.4±15.1			
Median (IqR)	100 (87.5 - 100)			91.7 (75.0 -100)			100 (86.4 -100)			

Suppuration (%)†										<0.0001s
Mean ±SD	13.9±17.6a			0.0±0.0b			10.8±19.0a			
Median (IqR)	10.4 (0.0 – 18.8)			0.0 (0.0 -0.0)			0.0 (0.0 – 12.5)			

PD (mm)†										0.288ns
Mean ±SD	3.5±1.4			3.6±1.4			3.5±1.3			
Median (IqR)	3.0 (3.0 – 4.0)			3.0 (3.0 -4.0)			3.0 (3.0 – 4.0)			
Sites (%) with PD per sextant*	≤3 mm	4-5 mm	≥6 mm	≤3 mm	4-5 mm	≥6 mm	≤3 mm	4-5 mm	≥6 mm	0.246ns
All sextants	422 (58)	250 (34)	54 (7)	394 (54)	265 (37)	67 (9)	386 (56)	254 (37)	44 (6)	
Upper Right	68 (57)	48 (40)	4 (3)	60 (56)	35 (32)	13 (12)	111 (64)	58 (33)	5 (3)	
Upper Anterior	73 (61)	39 (32)	8 (7)	98 (51)	73 (38)	21 (11)	37 (51)	29 (40)	6 (8)	
Upper Left	87 (50)	60 (34)	27 (16)	66 (61)	39 (36)	3 (3)	57 (56)	35 (34)	10 (10)	
Lower Left	67 (74)	22 (24)	1 (1)	36 (50)	34 (47)	2 (3)	30 (71)	12 (29)	0 (0)	
Lower Anterior	60 (56)	39 (36)	9 (8)	68 (60)	28 (25)	18 (16)	78 (50)	66 (42)	12 (8)	
Lower Right	67 (59)	42 (37)	5 (4)	66 (50)	56 (42)	10 (8)	73 (53)	54 (39)	11 (8)	
Width of KM (mm)†										0.089ns
Mean ±SD	3.8±1.5			3.7±1.6			3.9±1.7			
Median (IqR)	4.0 (3.0 – 5.0)			4.0 (2.0 – 5.0)			4.0 (3.0 – 5.0)			

Site (%) with KM*										0.231ns
≤2 mm	108 (21)			131 (25)			123 (24)			
>2 mm	411 (79)			391 (75)			387 (76)			

* 2;

† Kruskal-Wallis (different letters indicate significant difference. p<0.0001).

ns not statistically significant (p≥0.05); s statistically significant (p<0.05); IqR. (Interquartile range)

The multilevel regression analysis (Table 2) showed statistical significance for the variance at two levels (subject and site). Subject-level variance, considering all the pain measurements, was responsible for 65% of the total outcome variation. Site-level variance was associated with 30% of the variation of the results. Taking into consideration the subject level covariates, the factors of dental anxiety and periodontal diagnosis (localized or generalized periodontitis) had an influence (p<0.05) on the outcome, with at least two methods of pain intensity evaluation. The site-level covariates, suppuration and probing depth influenced (p<0.05) pain intensity on at least two of the measurement scales (p<0.05).

**Table 2:** Three-level random intercept model for the outcome variable of pain during scaling and root planing using the restricted maximum likelihood estimation (REML)

			Dependent Variables			
Fixed Effects	Pain Frequency		NRS-101		VRS-4	
	Estimate (SE)	p value	Estimate (SE)	p value	Estimate (SE)	p value
Intercept*	0,085	0.848ns	2,367	0.214ns	0,907	0.267ns
	(-0.442)		(-1.804)		(-0.817)	
Subject-level covariates						
Age	0,005	0.041s	-0,015	0.115ns	-0,002	0.107ns
	(-0.002)		(-0.009)		(-0.001)	
Gender	-0,2	0.001s	0,381	0.097ns	0,058	0.051ns
	(-0.062)		(-0.229)		(-0.03)	
Periodontal diagnosis	0,319	<0.0001s	0,182	0.336ns	0,252	<0.0001s
	(-0.051)		(-0.189)		(-0.024)	
Anxiety	0,024	0.005s	0,084	0.007s	0,008	0.054ns
	(-0.008)		(-0.031)		(-0.004)	
						
Sextant-level covariates						
Number of teeth	-0,039	0.158ns	-0,243	0.017s	0,012	0.672ns
	(-0.027)		(-0.101)		(-0.033)	
Position	0,005	0.948ns	0,245	0.522ns	-0,006	0.724ns
	(-0.072)		(-0.35)		(-0.013)	
						
Site-level covariates						
Dental plaque	0,0225	0.662ns	-0,036	0.851ns	-0,055	0.027s
	(-0.052)		(-0.192)		(-0.025)	
Bleeding on Probing	-0,106	0.107ns	-0,322	0.187ns	-0,092	0.004s
	(-0.066)		(-0.244)		(-0.032)	
Suppuration	0,217	0.008s	0,716	0.019s	0,074	0.062ns
	(-0.082)		(-0.304)		(-0.039)	
Probing Depth	0,007	0.667s	0,14	0.022s	0,02	0.011s
	(-0.016)		(-0.061)		(-0.008)	
Keratinized Mucosa	-0,029	0.140ns	0,081	0.267s	0,004	0.652ns
	(-0.019)		(-0.073)		(-0.009)	
						
Random effects						
Subject level	0,316	<0.0001s	5,523	<0.0001s	0,106	<0.0001s
	(-0.074)		(-1.294)		(-0.025)	
Sextant level	0,054	0.121ns	0,415	0.124ns	0,005	0.124ns
	(-0.035)		(-0.270)		(-0.003)	
Site level	0,246	<0.0001s	2,107	<0.0001s	0,036	<0.0001s
	(-0.008)		(-0.065)		(-0.011)	

* Formal significance of the intercept coefficient estimate remains trivial in the presence of covariates.

ns not statistically significant (p≥0.05);

s statistically significant (p<0.05)

Table 3 presents a comparison of the mean (±SD) hemodynamic parameters (secondary outcomes) of the different groups. Systolic blood pressure (Sys. BP), diastolic blood pressure (Dia. BP) and heart rate (HR) were assessed at distinct moments of the appointments, i.e., at baseline, during the procedure, and after the procedure; oxygen saturation (SpO_2_) was checked at baseline and after treatment. The results suggest that this type of intra-pocket anesthetic gel for periodontal SRP did not interfere with the hemodynamic indices.

**Table 3:** Comparison of mean (±SD) values of hemodynamic parameters

Parameters	Variables		Groups		p value*
		Placebo	lidocaine/prilocaine thermosetting gel (Oraqix^®^)	liposomal lidocaine/prilocaine thermosetting gel	
Sys. BP, mm Hg	Baseline	129.7±17.72	129.5±17.98	130.0±21.37	0.967ns
	During treatment	130.4±18.03	131.1±18.84	130.2±19.53	0.919ns
	After treatment	130.7±20.40	128.6±15.90	133.2±21.10	0.198ns
	p value*	0.821ns	0.314ns	0.326ns	- - -
	CV of Sys BP (%)	4.79±3.89	4.25±3.27	4.23±3.75	0.692ns
Dia. BP, mm Hg	Baseline	85.66±12.97	84.37±10.08	85.37±14.84	0.766ns
	During treatment	85.84±11.41	84.26±10.58	86.11±13.15	0.411ns
	After treatment	84.58±13.68	85.37±11.95	86.63±12.60	0.519ns
	p value*	0.346ns	0.386ns	0.653ns	- - -
	CV of Dia BP (%)	5.94±5.84	5.25±4.68	5.56±4.69	0.811ns
HR, beats/min	Baseline	93.92±18.63	93.16±17.30	95.58±19.38	0.449ns
	During Treatment	92.55±23.16	93.39±16.70	93.29±24.57	0.925ns
	After treatment	92.37±19.17	92.97±18.56	95.45±19.97	0.180ns
	p value*	0.704ns	0.923ns	0.518ns	- - -
	CV of HR (%)	5.44±4.10	4.13±3.17	5.19±4.79	0.322ns
SpO_2_, %	Baseline	95.26±1.97	95.53±1.67	95.05±3.05	0.331ns
	After treatment	95.66±1.76	95.55±1.57	95.26±1.88	0.179ns
	p value‡	0.347ns	0.907ns	0.564ns	- - -
	CV of SO_2_ (%)	0.65±0.53	0.72±0.73	0.81±1.61	0.708ns

Sys. BP, systolic blood pressure, Dia. BP, diastolic blood pressure, HR, heart rate, SpO_2_, oxygen saturation. CV, coefficient of variation

* Repeated measures ANOVA;

‡ Paired t test

ns not statistically significant (p≥0.05); s statistically significant (p<0.05)

The results showed that the volume of gel applied, the number of applications, the need for additional (rescue) anesthesia, the time required to perform SRP during each session, the difficulties during periodontal treatment, and the discomfort caused by periodontal therapy were similar between the groups, as were the volunteers' perceptions of the gel's flavor, with no statistically significant differences between intervention groups (Table 4).

**Table 4:** Data obtained in trans- and postoperative scaling and root planing in the three intervention groups

Parameters	Intervention Groups			p value
	Placebo	Oraqix^®^	Liposomal	
Anesthetic gel volume (mL)*				
Mean ±SD	0.54±0.24	0.55±0.29	0.53±0.28	0.754ns
Median (Interquartile range)	0.5 (0.37 – 0.72)	0.5 (0.40 – 0.60)	0.4 (0.30 – 0.62)	
Number of applications‡				
Mean ±SD	1.61±0.79	1.40±0.64	1.45±0.76	0.396ns
Median (Interquartile range)	1.0 (1.0 – 2.0)	1.0 (1.0 – 2.0)	1.0 (1.0 – 2.0)
Need for additional (rescue) anesthesia (%)†				0.250ns
No	30 (79)	35 (92)	31 (82)
Yes	8 (21)	4 (8)	7 (18)	
Time required for scaling and root planing (min)*				0.719ns
Mean ±SD	15.03±6.02	14.13±5.40	14.05±5.94	
Median (Interquartile range)	14.0 (10.7 – 19.0)	13.0 (10.0 – 17.0)	14.0 (8.0 – 18.0)	
Difficulties during periodontal therapy†				0.066ns
No	34 (89)	37 (97)	38 (100)	
Yes	4 (11)	1 (3)	0 (0)	
Discomfort due to periodontal therapy†				0.355ns
No	28 (74)	30 (79)	33 (87)	
Yes	10 (26)	8 (21)	5 (13)	
Volunteers' perceptions of the gel's flavor†				0.722ns
Acceptable	28 (74)	26 (69)	27 (71)	
Slightly unpleasant	10 (26)	10 (26)	10 (26)	
Very unpleasant	0 (0)	2 (5)	0 (0)	
I would not like to receive it again	0 (0)	0 (0)	0 (0)	

* ANOVA;

‡ Kruskal-Wallis;

† 2 .

ns not statistically significant (p≥0.05);

s statistically significant (p<0.05)

## Discussion

The results of this study did not show statistically significant differences between intervention groups regarding pain and discomfort; therefore, the null hypothesis was accepted. Nevertheless, a recent systematic review concluded that topical anesthesia is superior to a placebo during probing and SRP because it reduces the risk and intensity of pain, as well as the need for rescue anesthesia.^25^ However, there are many differences between studies regarding intra-pocket anesthesia, such as study design;^8^^,^^16^ composition of the anesthetic gel;^10^^,^^11^^,^^26^ use of occluded anesthesia;^26^ numerically small point estimate differences between treatments;^27^ and type of intervention.^14^ Most of these studies did not consider variables such as the influence of acoustic or sound stimuli on the patients' perception of pain, anxiety and fear;^5^^,^^6^ the possible discomfort caused by swallowing the gel during the procedures;^10^^,^^28^^,^^29^ as well as short application time (30 seconds to 2 minutes),^8^^,^^11^^,^^14^^,^^16^ removal of the gel during scaling and root planing, and dentin hypersensitivity during periodontal therapy.^2^

Our study had a cross-over design: all 38 volunteers were submitted to SRP using the three different gels (placebo and anesthetic) in different sextants. Sextant distribution and number of teeth per sextant were equivalent between intervention groups. The volunteers included in the study had similar periodontal parameters between sextants, including dental plaque, bleeding on probing, probing depth and width of the keratinized mucosa. Conversely, we found statistical differences regarding the percentage of suppuration. No suppuration sites were observed in the Oraqix^®^ group; in contrast, suppuration sites were present in 14% of the placebo group and 11% of the liposomal gel group. Suppuration was associated with higher pain frequency/intensity (NRS-101) during scaling and root planning (multilevel regression analysis). With regard to the issue of probing depth, it was associated with a higher level of pain intensity (NRS-101 and VRS-4); our results can be compared with those of other studies^10^^,^^13^^,^^14^ that had similar characteristics such as sample size and study design.

The association of local anesthetics with liposomal formulations increases the duration of anesthesia, decreases central nervous and cardiac toxicity, and decreases circulating plasma levels.^17^^–^^19^ Liposomal topical anesthetics provide a duration of approximately 10 min of anesthesia in the gingiva and buccal mucosa, results similar to those obtained by lidocaine-prilocaine cream.^21^^,^^22^ However, other factors could reduce the effectiveness of these local anesthetics, such as the possibility of removing the gel during scaling and root planing while using periodontal curettes or sonic/ultrasonic instruments; the presence of inflammation in the periodontal pockets, which can modify the anesthetics' pharmacological activity;^30^ and the inability of keeping the periodontal pocket internally dry due to the continuous plasma transudate generated from a gingival trauma caused by scaling and root planing.^31^ In our study, volunteers did not report transitory side effects due to the use of different topical anesthetics.

This study is the first to evaluate hemodynamic parameters (systolic blood pressure, diastolic blood pressure, heart rate and oxygen saturation) and anxiety during SRP using non-invasive anesthesia. No statistically significant differences were found between groups, suggesting that lidocaine/prilocaine intra-pocket anesthetic gels do not interfere with these parameters. Anxiety and fear can have a pain-increasing effect, creating an intentional bias towards the painful stimuli or pain-related sensation. Anxiety-induced somatic changes may occur from the activation of the hypothalamus-pituitary-adrenal axis, the main result being increased secretion of cortisol. Endogenous or exogenous epinephrine may cause or contribute to hemodynamic and cardiac changes.^32^ Dental anxiety may have an impact on the effect of local anesthesia, blood pressure, and oxygen saturation; it is significantly associated with increased heart rate.^2^^,^^33^ Lidocaine-prilocaine cream may cause methemoglobinemia and change arterial blood saturation and pulse oximetry.^34^ On the other hand, the lidocaine-prilocaine concentration observed after the intra-pocket application of Oraqix^®^ was below the threshold levels for toxic effect.^15^ Our study found a significant association between dental anxiety and pain, indicating that the use of non-invasive anesthesia might be a good option for anxious patients who have had previous negative experiences with conventional anesthesia.^35^

Some authors have reported that the use of split-mouth and cross-over designs significantly increased efficiency in statistical testing;^14^^,^^23^ however, others have argued that a parallel group study design would be more appropriate for this type of research in order to avoid the possibility of a carry-over or spill-over effects caused by the volunteers' perception of pain.^8^^,^^23^ Even though a parallel group design might contribute to subject blinding,^8^ it could be influenced by factors such as age, gender, ethnicity, previous pain experience and education.^14^ Our results showed that around 18% to 39% of the volunteers reported severe pain, suggesting that SRP can be a painful procedure for some patients. However, such findings can be widely divergent because they are influenced by factors such as age, type of periodontal therapy, and gender.^2^^–^^4^ In similar studies, some patients submitted to SRP using a placebo or anesthetic gel asked for rescue anesthesia, implying that this type of gel has limited applications.^8^^,^^11^^,^^16^^,^^36^

In our study, pain and discomfort were assessed during/after SRP using pain frequency and two pain-rating scales: a numerical rating scale (NRS-101) and a four-point verbal rating scale (VRS-4). Pain frequency provides similar results in comparison to the pain scale.^3^^,^^4^ The NRS-101 and VRS-4 scales are widely applied due to their validity, reliability and sensitivity;^37^^,^^38^ nevertheless, they have some limitations linked to the patients' age and education level, as well as to their difficulty expressing pain using numbers.^39^ In other similar studies using non-invasive periodontal anesthesia, the most common rating scales used were VRS and the visual analogue scale (VAS).^7^^–^^9^^,^^11^^,^^13^^,^^16^^,^^26^^,^^35^ Although the latter is considered more sensitive, it has also been claimed that it is not as easy to understand as the NRS-101 and VRS-4 scales, which could lead to higher failure rates.^37^ It is important to note that there is no ideal scale for measuring pain and the results need to be carefully interpreted by researchers.^39^

## Conclusion

In conclusion, we did not find differences between intervention groups in relation to pain frequency/intensity (primary outcome). The use of intra-pocket anesthetic gel for periodontal SRP did not interfere with the hemodynamic parameters (secondary outcome). Our results suggest limited indications for the use of non-invasive periodontal anesthesia: firstly, because periodontal procedures usually cause low or moderate pain, and secondly, because patients sometimes prefer not to receive local anesthesia. Nevertheless, some patients may experience severe pain during non-surgical periodontal therapy, and conventional local anesthesia is often necessary. The use of an intra-pocket anesthetic gel could be a good option for maintenance patients, anxious patients, or those who have a fear of needles. This is the first study to evaluate liposomal, thermosetting anesthetic gel during SRP. Consequently, further studies should be performed to verify its application in dental practice.
